# Altered Intrinsic Coupling between Functional Connectivity Density and Amplitude of Low-Frequency Fluctuation in Mild Cognitive Impairment with Depressive Symptoms

**DOI:** 10.1155/2018/1672708

**Published:** 2018-05-29

**Authors:** Xiaozheng Liu, Jiuzun Chen, Bangli Shen, Gang Wang, Jiapeng Li, Hongtao Hou, Xingli Chen, Zhongwei Guo, Chuanwan Mao

**Affiliations:** ^1^Department of Radiology, China-USA Neuroimaging Research Institute, The Second Affiliated Hospital and Yuying Children's Hospital, Wenzhou Medical University, Wenzhou, Zhejiang 325027, China; ^2^Department of Pain, The Second Affiliated Hospital and Yuying Children's Hospital, Wenzhou Medical University, Wenzhou, Zhejiang 325027, China; ^3^Tongde Hospital of Zhejiang Province, Hangzhou, Zhejiang 310012, China

## Abstract

Neuroimaging studies have demonstrated that major depressive disorder increases the risk of dementia in older individuals with mild cognitive impairment. We used resting-state functional magnetic resonance imaging to explore the intrinsic coupling patterns between the amplitude and synchronisation of low-frequency brain fluctuations using the amplitude of low-frequency fluctuations (ALFF) and the functional connectivity density (FCD) in 16 patients who had mild cognitive impairment with depressive symptoms (D-MCI) (mean age: 69.6 ± 6.2 years) and 18 patients with nondepressed mild cognitive impairment (nD-MCI) (mean age: 72.1 ± 9.7 years). Coupling was quantified as the correlations between the ALFF values and their associated FCDs. The results showed that the ALFF values in the D-MCI group were higher in the left medial prefrontal cortex (mPFC) and lower in the right precentral gyrus (preCG), and the FCD values were higher in the left medial temporal gyrus (MTG) than those in the nD-MCI group. Further, correlation analyses demonstrated that, in the D-MCI group, the mPFC was negatively correlated with the MTG. These findings may relate to the characteristics of mood disorders in patients with MCI, and they offer further insight into the neuropathophysiology of MCI with depressive symptoms.

## 1. Introduction

Mild cognitive impairment (MCI) is a neurological disorder that is associated with minimal cognitive impairments that are beyond those expected based on a person's age and education, but the changes are not severe enough to interfere with daily living, and they do not meet the criteria for dementia [1]. The conversion rate from MCI to dementia is approximately 12–16% per year [2]. Depression is a common symptom among individuals with MCI, with a prevalence of 32% [3].

Gao et al. [4] reported that depression was a major risk factor for the incidences of dementia and MCI. Further, recent meta-analyses showed that depressive symptoms increase the risk of MCI progressing to dementia and that depressive symptoms predict conversion from any type of MCI to all-cause dementia [5]. Neuroimaging studies have demonstrated that structural abnormalities in several brain regions are involved in the pathological process of depressive symptoms in MCI. Xie et al. [6] reported that depressive symptoms in MCI are related to grey matter volume loss in several brain regions, including the dorsal cingulate cortex, orbitofrontal cortex (OFC), ventromedial prefrontal cortex (vmPFC), posterior middle temporal gyrus (pMTG), and insula. Sacuiu et al. and Gonzales et al. [7, 8] also reported that MCI with depressive symptoms showed increased cortical atrophy in the anterior cingulate and the frontal lobe. Studies have also shown that, compared with those with nondepressed MCI (nD-MCI), patients with MCI and depression (D-MCI) have more white matter atrophy and white matter microstructural disruptions in the frontal, parietal, and temporal brain regions, especially in the hippocampal cingulum and fornix tracts [9–11]. Recently, researchers have used resting-state functional magnetic resonance imaging (R-fMRI) technology to study the functional changes in the brains of people with D-MCI. By analysing functional connectivity (FC), Zheng et al. [12] reported that, compared with nD-MCI, D-MCI was associated with higher effective connectivity between the right amygdala and the right lingual gyrus, right calcarine gyrus, and bilateral supplementary motor areas. Xie et al. [13] reported that, for people with MCI, scores on the Geriatric Depression Scale were positively correlated with functional connectivity in the network connecting the bilateral posterior cingulate cortex (PCC), middle temporal gyrus, and left dorsolateral prefrontal cortex (DLPFC). By analysing the amplitude of low-frequency fluctuations (ALFF), Li et al. [14] found that abnormal ALFF values in the left inferior frontal gyrus and left precuneus could effectively differentiate nD-MCI from D-MCI. Currently, the preliminary results showed that the affective network and the default mode network might be simultaneously damaged in patients with D-MCI.

FC and ALFF are two fundamental fMRI parameters. FC characterises the degree of synchronisation between low-frequency fluctuations in the resting brain and requires the definition of a relation (e.g., Pearson correlation) between the features of different voxels. Therefore, FC describes network properties, while the ALFF represents the amplitude of resting-state spontaneous brain activity by calculating the voxel-wise total power of a given fMRI time course within the low-frequency band [14]. These two measures have been shown to have a close relationship [15–17] and to be altered within the affective network and the default mode network in both MCI and major depressive disorder [12, 13, 18]. Compared with healthy elderly subjects, AD and MCI patients show absent FC density-ALFF coupling in the anterior and posterior cingulate cortex and the temporal cortex [15]. Similar methods have been used to observe changes in functional brain features in erythaematosus and epilepsy patients [16, 17]. Thus, we believe that the coupling patterns between these two parameters may provide a new measure that can help us understand the underlying neuropathophysiology of D-MCI and enhance its identifiability by resting-state fMRI.

We, therefore, utilised the measures of ALFF and FC density (FCD), a global and voxel-wise measure of FC, to investigate alterations in amplitude-connectivity coupling in D-MCI. Given the common differences that have been reported in the brain networks of patients with D-MCI [13–17], we hypothesised that those with D-MCI would have altered brain fluctuations in brain regions associated with cognitive and emotional regulation.

## 2. Materials and Methods

### 2.1. Patients

Eighteen patients with nD-MCI and 16 with D-MCI were recruited from the outpatient department of Tongde Hospital in Zhejiang Province, China, from July 2013 to August 2016. The study was approved by the local ethics committee, and all participants gave their written informed consent before the MR scanning. All participants were right-handed, and the groups did not significantly differ in age, sex, or years of education.

A diagnosis of MCI includes memory impairment that does not meet the criteria for dementia. The criteria for MCI [19] were as follows: (a) impaired memory performance on a normalised, objective verbal memory test; (b) recent history of symptomatic worsening in memory; (c) normal or near-normal performance (score > 24) on the Mini-Mental State Examination (MMSE), as well as on the activities of daily living scale (score > 24); (d) a global rating of 0.5 on the clinical dementia rating scale; and (e) the absence of dementia.

Depressive symptoms were identified by professional psychiatrists according to the *Diagnostic and Statistical Manual of Mental Disorders*, fourth edition [20]. The severity of depressive symptoms was evaluated using two clinical scales: the Hamilton Depression Rating Scale (HAMD) [21] and the Neuropsychiatric Inventory (NPI) [22]. We considered HAMD scores ≥ 7 and NPI scores ≥ 4 in the depression domain to be clinically significant [23].

Patients were excluded if they had a history of psychiatric disorders, alcohol or substance abuse/dependence during the prior five years, MMSE scores < 24, a history of neurological disease, MRI contraindications, or unstable chronic medical conditions.

### 2.2. MRI Scan

All scanning was collected using an 8-channel head coil in a 3T Siemens scanner (Siemens Magnetom Verio; Siemens Medical Systems, Erlangen, Germany) at Tongde Hospital in Zhejiang Province. All patients were asked to keep their heads still and their eyes closed during image acquisition. T1-weighted high-resolution anatomical images were acquired using a 3D magnetisation-prepared rapid gradient echo sequence with the following parameters: repetition time = 1900 ms, echo time = 3.44 ms, inversion time = 900 ms, flip angle = 9°, 128 sagittal slices, field of view = 256 mm, and slice thickness = 1 mm. Functional resting-state fMRI images were acquired using a T_2_^∗^-weighted echo-planar imaging sequence with the following parameters: 33 axial slices, thickness/gap = 4.8/0 mm, in-plane resolution = 3.4 × 3.4 mm^2^, repetition time = 2000 ms, echo time = 30 ms, flip angle = 90°, and field of view = 200 mm. Each condition lasted for 6 min 40 s, and 200 functional volumes were obtained.

### 2.3. Data Preprocessing

All fMRI data were preprocessed using SPM8 (http://www.fil.ion.ucl.ac.uk/spm) and Data Processing Assistant for Resting-State fMRI (http://www.restfmri.net). The first ten time frames were discarded to ensure an MR steady state. The preprocessing steps comprised slice-timing correction for interleaved acquisitions, 3D motion correction, linear drift removal, spatial smoothing with a Gaussian smoothing kernel (full width at half maximum = 6 mm), and spatial normalisation to the standard Montreal Neurological Institute (MNI) brain space with a resampling resolution of 3 × 3 × 3 mm^3^. Subjects were discarded if head motion exceeded 1.5 mm translation in x, y, or z directions or 1.5° of maximum rotation about the three axes. All remaining smoothed images were filtered using a typical temporal bandpass (0.01–0.08 Hz) to reduce low-frequency drift and physiological high-frequency respiratory and cardiac noise. Linear trends were also removed within each time series.

### 2.4. ALFF Calculation

ALFF was calculated using the REST software (http://www.restfmri.net). Briefly, for a given voxel, the time series was first converted to the frequency domain using a fast Fourier transform. The square root of the power spectrum was computed and then averaged across 0.01–0.08 Hz. This averaged square root was taken as the ALFF at the given voxel [24]. Then, the ALFFs were standardised by dividing by the whole-brain average of the ALFF at each voxel, which measures the absolute strength or intensity of spontaneous low-frequency oscillations.

### 2.5. FCD Calculation

We performed voxel-based whole-brain correlation analysis on the preprocessed R-fMRI data, as has been well described in previous studies [25]. Pearson's correlation coefficients (*r*) were computed between the time series for all pairs of grey matter voxels within a grey matter mask. The FCD for a given voxel was calculated as the sum of the significant connections between a given voxel and all other voxels by thresholding each correlation at *r* > 0.25. Finally, to improve the normality of the data, the voxel-wise FCD values were transformed into a *Z*-score map using a Fisher *Z* transformation. Because of the uncertain interpretation and the detrimental effects on test-retest reliability, only positive correlations were considered in the FCD calculations.

### 2.6. Statistical Analysis

To examine ALFF and FCD differences between the groups, a two-sample *t*-test was performed between the two groups using REST. The 3dClustSim program, which is based on Monte Carlo simulation and implemented in AFNI (http://afni.nimh.nih.gov), was used for multiple comparison correction [26]. The statistical threshold was set at *P* < 0.005 and cluster size > 28 voxels, which corresponded to a corrected *P* < 0.05.

### 2.7. Coupling of ALFF and FCD

To test the proposal that alterations in amplitude-connectivity coupling in D-MCI and, thus, to differentiate D-MCI from nD-MCI, we computed the coupling between the FCD and ALFF across subjects in each group. Based on the two-sample *t*-test results of ALFF and FCD, subject-specific ALFF and FCD values were first extracted from the abnormal brain regions, respectively. Then, we performed a voxel-by-voxel Pearson correlation analysis between ALFF and FCD values in regions with alterations (between amplitude and FC) in each group, respectively.

## 3. Results

### 3.1. Neuropsychological Results

Age (*t* = 0.898, *P* = 0.376), sex distribution (*χ*^2^ = 0.161, *P* = 0.735), and years of education (*t* = 0.464, *P* = 0.645) did not differ between the two groups. Detailed demographics and the corresponding statistical tests are presented in Table 1.

### 3.2. ALFF Results

**Figure 1:**
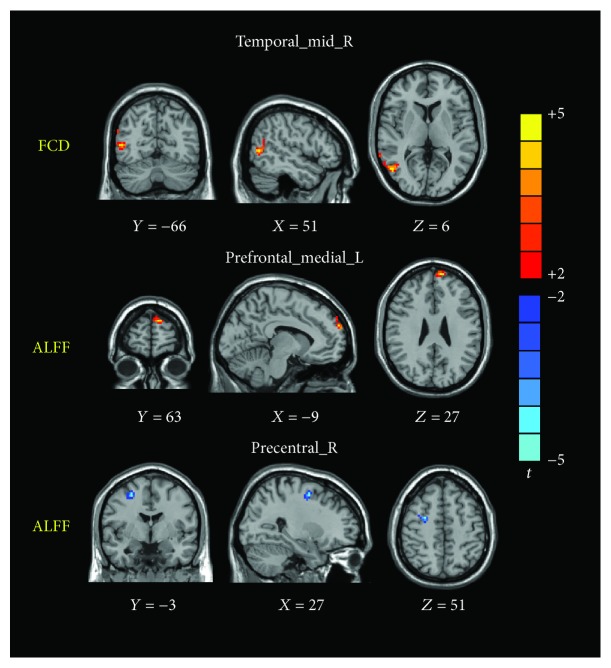
Brain regions showing differences in FCD or ALFF values between the D-MCI and nD-MCI groups (contrast = D-MCI − nD-MCI).

The two-sample *t*-tests revealed several related brain regions in which the ALFF values differed significantly between the D-MCI and nD-MCI groups (*P* < 0.005, 3dClustSim-corrected; Table 2). In particular, we found that the D-MCI group exhibited significantly higher ALFF values than the nD-MCI group in the right precentral cortex and significantly lower ALFF values in the left medial prefrontal cortex (Figure 1).

### 3.3. FCD Results

Similar to the ALFF values, the two-sample *t*-tests revealed significant differences in FCD between the D-MCI and nD-MCI groups (*P* < 0.005, 3dClustSim-corrected; Table 2). Specifically, we found that FCD values in the right middle temporal gyrus were greater in those with D-MCI than in those with nD-MCI (Figure 1).

### 3.4. Altered Coupling between ALFF and FCD

**Figure 2:**
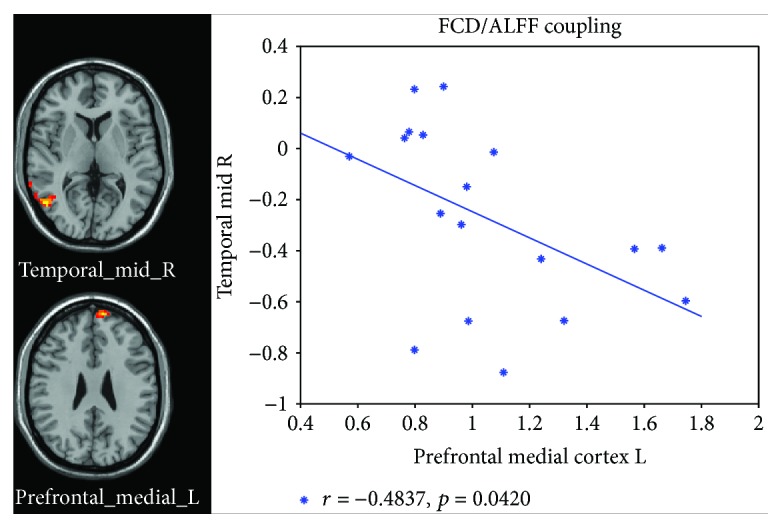
Significant correlations between FCD and ALFF in the nD-MCI brain. FCD values of the right temporal middle gyrus and ALFF values of the medial prefrontal cortex were subtracted from the abnormal brain regions resulting from *t*-contrast nD-MCI versus D-MCI.

Correlation analyses between the abnormal ALFF and FCD brain regions revealed a negative correlation between mPFC and MTG coupling in the nD-MCI (Figure 2) but not in the D-MCI group. Thus, for patients with nD-MCI, coupling was high in the mPFC when it was low in the MTG and vice versa.

## 4. Discussion

Here, we investigated alterations in ALFF-FCD and coupling of ALFF with FCD in patients with D-MCI and nD-MCI. The D-MCI group exhibited significantly higher FCD in the right MTG, significantly higher ALFF in the left mPFC, and significantly lower ALFF in the right precentral gyrus (preCG) than the nD-MCI group. We also found a negative correlation between ALFF-FCD coupling in the mPFC and the MTG but only in patients with D-MCI.

The mPFC is an important node in the cortico-striato-pallido-thalamic loops and in the medial network, contributing to emotional processes and regulation [27]. Several neuroimaging studies have demonstrated that the mPFC is a key brain region in depressive symptomatology. Xie et al. [6] reported that depressive symptoms in MCI are related to grey matter volume loss in several brain regions, including the mPFC, posterior MTG, and insula. Furthermore, Sacuiu et al. and Gonzales et al. reported that D-MCI patients have increased cortical atrophy in the anterior cingulate and the frontal lobe, and decreased left mPFC thickness was associated with increased negative affect [7, 8]. Other studies have also demonstrated that depressed individuals with MCI had more white matter atrophy in the frontal, parietal, and temporal cortices than those with no-symptom MCI did [9–11]. Based on R-fMRI and ALFF analyses, Wang et al. [28] found that MDD patients had abnormal ALFF values in the mPFC, precentral gyrus, and other regions. Using magnetic resonance spectroscopy, McEwen et al. [29] reported that glutamate levels in the mPFC were higher in women with postpartum depression than in controls. Savitz et al. [30] found that dysfunction of the serotonin-1A receptor in the mPFC might play a role in the genesis of MDD. Additionally, a recent animal study [31] suggested that synaptogenic activity in the mPFC is associated with a rapid antidepressant response to ketamine. Thus, our results are in line with previous findings, suggesting that the altered spontaneous ALFF values in the mPFC might be a characteristic of the neurological impairments of MCI with depressive symptoms.

A neuroimaging study reported that the preCG was involved in MDD [32]. Peng et al. [33] observed decreased cortical thickness in the preCG in patients with untreated first-episode MDD. Using voxel-based morphometry, Taki et al. [34] found that male patients with subthreshold depression had significantly smaller right preCG volumes than healthy controls. Several studies have also demonstrated that grey matter structural changes in the right preCG can predict the subsequent onset of MDD and pose an increased risk for mood disorders [35–37]. A study by Ho et al. [38] revealed that the symptom of alexithymia that occurs in MDD was associated with reduced functional connectivity in the right preCG and several other right hemisphere regions that are associated with cognitive regulation in the default mode network (DMN). They found that subjective pleasantness was related to the preCG, right cerebellum, and right inferior frontal gyrus [39]. The preCG is located at the primary motor cortex, which is required for the initiation of purposeful movements via integration of information sent from the sensorimotor cortex [40]. Several studies have confirmed the association between psychomotor retardation, poor action planning, and alterations in the preCG [41, 42]. Therefore, the abnormal ALFF in the preCG of our study might be related to the depressive symptoms in our D-MCI patients.

MTG is an important node within the DMN and the affective network (AN), which are involved in self-referential processing, emotion, and regulation [43–45]. Neuroimaging evidence has demonstrated that altered FC occurs in MDD patients between the MTG and other nodes within the DMN and AN. For the FC of the right MTG, Ma et al. [46] found that patients with treatment-resistant depression as well as those with treatment-responsive depression showed abnormal connectivity mainly in the DMN. Based on FC analyses of the subgenual anterior cingulate cortex (sgACC), MDD patients showed disruptions in FC between the sgACC and MTG [47]. Du et al. [48] investigated the brain-circuit mechanisms of suicidal ideation (SI) in MDD, finding that the SI group exhibited decreased intrinsic FC among the rostral anterior cingulate cortex (rACC), the orbitomedial prefrontal cortex, and the right MTG compared with healthy controls and those with MDD without SI. In the SI group, the FC strength between the right rACC and the MTG positively correlated with SI severity. The altered FCD in the MTG in the present study expands the knowledge gained from previous studies of abnormal FC in MDD patients by investigating the FC in the right MTG. MDD is characterised by the presence of negative thoughts about oneself, the world, or the future. We hypothesised that the altered FC in the right MTG might contribute to the negative thoughts and negative emotions observed in patients with MCI and depressive symptoms.

Interestingly, we found a negative correlation between ALFF-FCD coupling in the mPFC and the MTG but only in the nD-MCI group. De Bellis and Hooper [49] reported that maltreated youth with depressive disorders had significantly weaker activation of the mPFC in response to attentional targets and stronger activation in the MTG in response to sad distracters during an emotional oddball task than controls. They concluded that maltreated youth with depressive disorders had dysfunctional cognitive and emotional processing because of the mPFC involvement in cognitive control circuits and the MTG involvement in ventral emotional circuits. Du et al. [48] also reported that depressed patients with suicidal ideation had decreased intrinsic FC between the mPFC and the right MTG, and they suggested that the disrupted frontolimbic circuits might impact decision-making and emotional processing in depressed patients with suicidal ideation. The mPFC and right MTG are two nodes within the DMN. According to previous neuroimaging studies, the DMN can be generally divided into anterior (aDMN) and posterior (pDMN) subnetworks. The former is related to self-referential thought, and the latter is related to episodic memory retrieval and scene construction [50–52]. The aDMN mostly consists of the mPFC, anterior cingulate cortex, anterior temporal lobe, and inferior frontal gyrus, whereas the pDMN mostly contains the posterior cingulate cortex (PCC), precuneus, angular gyrus, hippocampus, and temporal lobe. A recent fMRI study revealed that the activation within these subsystems and the connectivity between the aDMN and pDMN contribute differently to future-oriented thoughts [53]. How the brain creates emotions is complex and esoteric. Using a psychological constructionist approach, Lindquist et al. hypothesised that several brain regions, including the mPFC, medial temporal lobe (MTL), and PCC, are important for realising instances of emotional experiences and perceptions [54]. The negative correlation in ALFF-FCD coupling between the mPFC and the MTG in our study might contribute to decision-making, future-oriented thoughts, and experiences and perceptions of emotion in MCI with depressive symptoms.

This study had several potential limitations. First, the sample size was relatively small, which might have resulted in low statistical power and the absence of correlations between FCD/ALFF and physiological measurements. Second, previous studies have suggested that neuronal oscillations in various frequency intervals have different specific properties and physiological functions [14]. While short- and long-range FCD have been used in fMRI studies [55], further investigations into coupling between long- or short-range FCD with ALFF at various frequency intervals should be performed. Finally, the choice of a clinical assessment scale is very important. In our study, we selected the HAMD and the NPI to evaluate depressive symptoms in MCI, as a previous study did [9]. However, other studies have used different scales, such as the Geriatric Depression Scale [56], the Cornell Scale for Depression in Dementia [57], and the Center for Epidemiologic Studies Depression Scale [58]. Evaluating depressive symptoms comprehensively and accurately in MCI is difficult because of the cognitive impairment. Therefore, in future studies, we should evaluate clinical depressive symptoms with multiple scales, based on the characteristics of each scale.

## 5. Conclusions

In the current study, we observed differences between D-MCI and nD-MCI in terms of FCD and ALFF values that were derived from R-fMRI. We also examined differences in ALFF-FCD coupling in D-MCI and nD-MCI. The findings indicated that pathological factors lead to dysfunctional ALFF-FCD coupling between the mPFC and the MTG in D-MCI. Investigation of imaging coupling provides a synergistic approach to unravelling the features of the functional changes in MCI and provides a new insight into the underlying neural mechanism of MCI with depressive symptoms.

## Figures and Tables

**Table 1:** Demographics and neuropsychological data.

	D-MCI group	nD-MCI group	*t*/*χ*^2^	*P* value
Gender, *n* (M/F)	16 (6/10)	18 (7/11)	0.007	1.000
Age, years	69.6 ± 6.2	72.1 ± 9.7	0.898	0.376
Education, years	8.3 ± 2.1	8.5 ± 1.8	0.464	0.645
MMSE	26.6 ± 1.1	26.6 ± 1.0	−0.037	0.971
HAMD	11.7 ± 3.1	0	16.0652	0.000
D-NPI	7.19 ± 2.3	0	13.3614	0.000

Data represent mean ± SD. Data were analysed using independent-samples *t*-tests. D-MCI: mild cognitive impairment with depression; nD-MCI: nondepressed mild cognitive impairment; M: male; F: female; MMSE: Mini-Mental State Examination; D-NPI: depression domain on the Neuropsychiatric Inventory; HAMD: Hamilton Depression Rating Scale.

**Table 2:** Brain regions with significantly lower ALFF-FCD values in the D-MCI group than in the nD-MCI group.

Brain regions	Voxels	BA	MNI coordinates			*t* value
			*X*	*Y*	*Z*	
*ALFF*						
Prefrontal_medial_L	32	10	−9	63	27	4.6674
Precentral_R	28	6	27	−3	51	−5.7117
*FCD*						
Temporal_mid_R	117	37	51	−66	6	4.9394

D-MCI: mild cognitive impairment with depression; nD-MCI: nondepressed mild cognitive impairment; MNI: Montreal Neurological Institute; BA: Brodmann area.
